# Ultra‐Permeable Single‐Walled Carbon Nanotube Membranes with Exceptional Performance at Scale

**DOI:** 10.1002/advs.202001670

**Published:** 2020-11-09

**Authors:** Melinda L. Jue, Steven F. Buchsbaum, Chiatai Chen, Sei Jin Park, Eric R. Meshot, Kuang Jen J. Wu, Francesco Fornasiero

**Affiliations:** ^1^ Department of Physical and Life Sciences Lawrence Livermore National Laboratory Livermore CA 94550 USA

**Keywords:** chemical resistance, enhancement factors, high‐density SWCNTs, large‐area CNT membranes, nanofiltration

## Abstract

Enhanced fluid transport in single‐walled carbon nanotubes (SWCNTs) promises to enable major advancements in many membrane applications, from efficient water purification to next‐generation protective garments. Practical realization of these advancements is hampered by the challenges of fabricating large‐area, defect‐free membranes containing a high density of open, small diameter SWCNT pores. Here, large‐scale (≈60 cm^2^) nanocomposite membranes comprising of an ultrahigh density (1.89 × 10^12^ tubes cm^−2^) of 1.7 nm SWCNTs as sole transport pathways are demonstrated. Complete opening of all conducting nanotubes in the composite enables unprecedented accuracy in quantifying the enhancement of pressure‐driven transport for both gases (>290× Knudsen prediction) and liquids (6100× no‐slip Hagen–Poiseuille prediction). Achieved water permeances (>200 L m^−2^ h^−1^ bar^−1^) greatly exceed those of state‐of‐the‐art commercial nano‐ and ultrafiltration membranes of similar pore size. Fabricated membranes reject nanometer‐sized molecules, permit fractionation of dyes from concentrated salt solutions, and exhibit excellent chemical resistance. Altogether, these SWCNT membranes offer new opportunities for energy‐efficient nano‐ and ultrafiltration processes in chemically demanding environments.

## Graphical abstract

Membranes with an ultrahigh density of open single‐walled carbon nanotubes (SWCNT) as the sole transport pathways are demonstrated at the unmatched scale of 60 cm^2^. These membranes enable accurate quantification of the massive SWCNT amplification of pressure‐driven flow, outperform state‐of‐the‐art commercial membranes with similar pore diameters, and display excellent oxidative resistance.

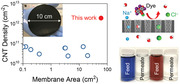

Carbon nanotubes (CNTs) have garnered sustained interest in many areas of material science due to their outstanding thermal, mechanical, electrical, and fluidic properties and have enabled advancements in a variety of functional materials and technologies.^[^ ^1^, ^2^ ^]^ In separation applications, CNTs are interesting membrane building blocks as their unique transport properties lead to exceptionally large flow rates compared to conventional porous materials with commensurate pore sizes.^[^ ^3^, ^4^, ^5^, ^6^ ^]^ This transport enhancement has been attributed to the atomic smoothness and hydrophobicity of CNT walls,^[^ ^6^, ^7^, ^8^ ^]^ as well as nanoconfinement‐induced structural changes^[^ ^9^ ^]^ and variations in hydrodynamic properties of the transporting fluid.^[^ ^10^ ^]^ Both computational and experimental studies have investigated pressure‐driven CNT flow enhancement, yet reported magnitudes span too wide a range of values to be useful for application‐driven design of CNT fluidic systems. On the computational side, these discrepancies in transport enhancement are linked to challenges associated with accurately predicting the confined fluid behavior^[^ ^11^ ^]^ and model/method‐dependent results.^[^ ^12^, ^13^ ^]^ On the experimental side, the primary limiting factors are creating leak‐free fluidic platforms and quantifying the number of open tubes. Accurately measuring the mass flow rate through a single, nanometer‐sized CNT is difficult^[^ ^14^ ^]^ because state‐of‐the‐art methods lack adequate sensitivity and small, yet comparatively significant leak pathways are harder to eliminate as the tube diameter decreases.^[^ ^7^ ^]^ Current methods to estimate the number of open tubes in a CNT composite membrane also suffer critical shortcomings. Indirect measurements such as KCl diffusion^[^ ^4^, ^15^, ^16^, ^17^, ^18^, ^19^ ^]^ rely on the unproven assumption of hindered or bulk transport^[^ ^20^ ^]^ inside the CNTs, whereas direct counting of nanotubes with imaging techniques is unable to distinguish between transporting and blocked tubes. Furthermore, tube entrance effects and boundary layer resistances are nonnegligible^[^ ^20^, ^21^ ^]^ in high flux membranes and can introduce errors estimating the nanotube number from transport rate measurements with salt solutions.

For most applications, outperforming commercially available products in both selectivity and transport rates requires large‐area membranes with a high density of small diameter CNTs. Many CNT membrane fabrication approaches have been attempted to address these demands.^[^ ^22^ ^]^ One common method utilizes dispersions of CNTs in polymer matrices and may include intermediate alignment steps to orient the nanotubes in the direction orthogonal to the membrane plane.^[^ ^16^, ^23^, ^24^, ^25^, ^26^, ^27^, ^28^, ^29^, ^30^, ^31^ ^]^ This strategy offers the advantage of independent control over the CNT/polymer source and is amenable to continuous, large‐scale fabrication techniques such as roll‐to‐roll manufacturing. Albeit a commercialization effort for one example of these membranes is already underway,^[^ ^23^ ^]^ CNT density and composite porosity are often too low to best state‐of‐the‐art membranes. Additionally, when used for fundamental studies of flow enhancement, the quantification of open tubes that span this type of CNT membrane is nontrivial. Alternative fabrication routes based on vertically aligned CNT (VACNT) “forests” provide better platforms for accurate determination of transport enhancement and produce the highest reported CNT pore densities and membrane transport rates to date.^[^ ^3^, ^4^, ^18^, ^21^, ^32^, ^33^ ^]^ However, fabrication at scale is more complex and economically less viable because the CNTs must be grown with the desired properties over the entire membrane area and subsequent processing must preserve the CNT alignment.

The work presented here addresses the key challenges related to membrane scale up, flow enhancement quantification, and performance improvement. We developed wafer‐scale membranes with a precisely quantified, high density of small‐diameter CNTs as the conductive pores. We leveraged our previous CNT synthesis achievements^[^ ^34^ ^]^ to produce single‐walled CNTs (SWCNTs) with diameters below 4 nm and densities exceeding 10^12^ tubes cm^−2^ on 100 mm silicon wafers. We demonstrate that 60 cm^2^ membranes made from these large‐area forests display the same transport and selectivity properties as 1 cm^2^ samples, indicating that a negligibly low defect density can be maintained with scale up. This sample size represents the largest VACNT membrane area proven to be defect‐free to date. Opening all the CNTs in the membrane to fluid flow enables us to accurately quantify the gas and liquid enhancement factors in SWCNTs, which are shown to be several times larger than previously reported in the literature for similar pore sizes. In addition, we demonstrate that these high‐flux membranes are promising for nano‐ and ultrafiltration applications, especially since they retain their transport properties after aggressive chemical treatments.

CNT composite membranes of ≈1 cm^2^ active area were used to benchmark the membrane transport and separation performance at various operating conditions, whereas large area, ≈60 cm^2^ membranes were employed to demonstrate high performance at scale (**Figure** **1**a). Composite membranes were fabricated by infiltrating high‐density, vertically aligned CNT arrays with vapor‐deposited parylene N as previously reported.^[^ ^20^, ^21^, ^32^ ^]^ The SWCNTs have an average diameter of 1.7 ± 0.7 nm (inner diameter 1.4 ± 0.7 nm) with all the tubes being less than 4 nm (Figure 1b). Excess polymer covering the nanotubes was removed with oxygen plasma treatment, which also uncaps the CNTs and creates the carboxylic acid groups on the graphitic surface.^[^ ^21^, ^35^, ^36^ ^]^ To monitor the percentage of open CNTs, we measured the nitrogen permeance periodically during the etching process. In Figure 1c, the first 2 µm of removed material corresponds to the excess polymer layer. As etching begins to reach the CNTs, the permeance first increases steeply and then plateaus at ≈3.5 × 10^−5^ mol m^−2^ s^−1^ Pa^−1^ when all the available CNTs have been opened. Continued etching does not appreciably increase the permeance until defects are created.

**Figure 1:**
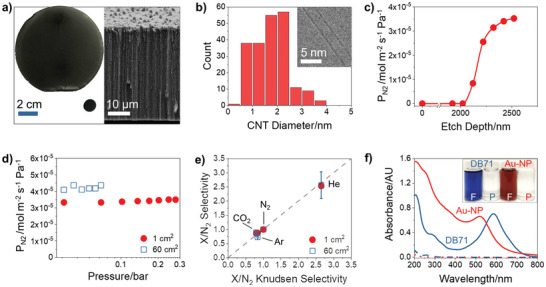
Fully opened SWCNT membranes. a) Pictures of small 1 cm^2^ and large 60 cm^2^ membranes before mounting (left) with SEM imaging of the composite membrane cross‐section (right). b) CNT diameter distribution determined by TEM with an average of 1.7 ± 0.7 nm (inner diameter 1.4 ± 0.7 nm). Inset: Representative TEM image of an SWCNT. c) Nitrogen permeance (P_N2_) increase during membrane opening with oxygen plasma etching. Line used to guide the eye. d) Nitrogen permeance measured at different applied pressures. e) Measured ideal gas selectivity (gas X relative to nitrogen, X/N_2_) of CNT membranes compared to predictions based on Knudsen diffusion. Parity line (*y* = *x*) included for comparison. Error bars represent the standard deviation of two to three membranes and are smaller than the data point in most cases. f) UV‐vis spectra of Direct Blue 71 (DB71) and 5 nm PEG‐coated gold nanoparticle (Au‐NP) solutions used in filtration experiments with a 1 cm^2^ membrane. Feed and permeate spectra are shown in solid and dashed lines, respectively. Inset: Pictures of feed (F, colored) and permeate (P, clear) solutions.

Both gas and liquid transport behaviors of our membranes provide evidence that transport is indeed through the CNT interiors and not through alternative leak pathways. Defects in the polymer matrix would be expected to increase in size with extended etching, leading to a continuous rise in the permeance. Larger defects would also manifest as increased permeance with applied pressure due to viscous flow contributions (Figure 1d). Instead, gas transported through our membranes with nanometer‐sized CNT pores follows Knudsen diffusion behavior and scales inversely with the square root of the molecular weight of the gas species. Indeed, Figure 1e shows the excellent agreement between the experimentally measured and predicted ideal gas pair selectivities, further suggesting a lack of large defects. Liquid‐phase experiments were also conducted to directly probe for smaller, nanometer‐sized defects and confirmed an absence of leaky transport pathways. Independent of the sample size, the membranes shown here reject more than 99% of both the negatively charged dye Direct Blue 71 (DB71) (3 × 1.5 × 1.5 nm^3^) and the neutral 5 nm diameter polyethylene glycol (PEG)‐coated, gold nanoparticles. Rejection of the dye is due to a combination of electrostatic repulsion and size exclusion, whereas size sieving alone completely prevents nanoparticle permeation. Additional rejection tests with an expanded set of gold nanoparticle sizes (1.8, 2.2, 3, 4, and 5 nm diameters; neutral polyvinylpyrrolidone and positively charged cetyltrimethylammonium bromide coatings) and high purity dyes (Erythrosin B, Eosin Y, Thymolphthalein, and m‐Cresol Purple) were performed with 1 cm^2^ sized membranes. Except for m‐Cresol Purple (95% rejection), all other probe molecules are completely rejected by the membranes. As a final defect assessment detailed in our separate work,^[^ ^20^ ^]^ control membranes with intentionally clogged CNT channels do not transport fluids even after extensive etching beyond the limit required to open membranes in this study. Altogether, these experiments conclusively prove that the measured transport is only through the CNT channels. This evidence of defect‐free transport relies on more stringent criteria than in previous CNT literature, since it excludes the presence of leaky pathways of any size, both larger and smaller than the CNT diameters.^[^ ^20^ ^]^ It is also important to note that both the 1 and 60 cm^2^ membranes display identical transport/selectivity behavior in the gas permeation and liquid rejection tests, thus demonstrating that the fabrication process can be scaled up without compromising performance.

We next utilized these fully open, defect‐free membranes to accurately quantify the pressure‐driven flow enhancement known to be displayed by both gases and liquids in CNTs.^[^ ^3^, ^15^, ^17^, ^18^, ^21^, ^23^, ^37^, ^38^, ^39^, ^40^ ^]^ In these calculations, we assumed that all the CNTs in the membrane are conducting fluids, and a detailed justification of this assumption is given in the Supporting Information. Nitrogen permeance was found to be 320 times greater than predicted from Knudsen diffusion through equally sized pores (**Figure** **2**a). The enhancement factors measured for several gases surpass previous literature values by a factor of 4–11 (Figure 2b), likely because a larger fraction of CNTs is accessible for transport.^[^ ^15^, ^17^, ^18^, ^23^, ^37^ ^]^ Although continuum fluid mechanics may not strictly apply to liquid flow through a few nanometer diameter pores, we follow the literature convention^[^ ^3^, ^15^, ^21^, ^38^, ^39^, ^40^ ^]^ of benchmarking transport rates in CNTs against the no‐slip Hagen–Poiseuille equation. We measured an astonishing 6100‐fold enhancement in the pure water permeance for our membranes. This value is close to the upper bound of the experimental data obtained by Holt et al.^[^ ^3^ ^]^ for 1.6 nm double‐walled CNTs and higher than other previously reported results for similar pore sizes (Figure 2d).^[^ ^3^, ^15^, ^21^, ^38^, ^39^, ^40^ ^]^ This exceptional performance is maintained even as the membrane area is increased by an order of magnitude (Figure 2a,c).

**Figure 2:**
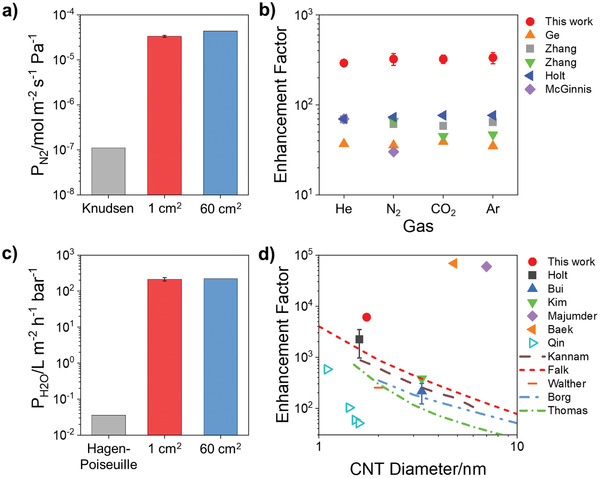
Transport through fully opened SWCNT membranes. a) Nitrogen permeance (P_N2_) for 1 and 60 cm^2^ SWCNT membranes compared to prediction based on Knudsen diffusion. Error bars represent the standard deviation of three membranes. b) Reported gas enhancement factors for CNT membranes with 7.7 (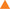),^[^ ^37^ ^]^ 7 (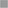,^[^ ^17^ ^]^ 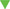),^[^ ^18^ ^]^ 1.6 (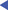),^[^ ^3^ ^]^ and 1.2 nm (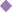)^[^ ^23^ ^]^ tube diameter compared to the 60 cm^2^ SWCNT membranes with 1.7 nm (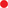) tube diameter from this work. Error bars represent the standard deviation of four membranes, both small and large. c) Pure water permeance (P_H2O_) for 1 and 60 cm^2^ SWCNT membranes compared to prediction based on no‐slip Hagen–Poiseuille flow. Error bars represent the standard deviation of three membranes. d) Pure water enhancement factors in CNTs from literature experiments (data points)^[^ ^3^, ^15^, ^21^, ^38^, ^39^, ^40^ ^]^ and simulations (lines)^[^ ^11^, ^13^, ^41^, ^42^, ^43^ ^]^ compared to our results for a 60 cm^2^ SWCNT membrane. Error bar for this work represents the standard deviation of four membranes (both small and large) and is smaller than the data point.

The ultra‐permeable membranes in this work exhibit the best co‐optimization of small CNT diameter, high tube density, and large membrane area reported so far. The exceptional structural properties and transport performance of our CNT membranes are highlighted by the comparison with literature values in **Figure** **3**. Although a previous report^[^ ^23^ ^]^ describes CNT composite membranes with areas as large as 570 cm^2^, selective transport was only demonstrated through a smaller 20 cm^2^ coupon. Thus, to the best of our knowledge, our work describes the largest SWCNT membrane to date that has been proven to be defect‐free. Moreover, our high density of open CNTs allows for a pure water permeance in excess of 200 L m^−2^ h^−1^ bar^−1^ at wafer scale, substantially higher than commercial nano‐ and ultrafiltration membranes of similar pore sizes. This result further demonstrates that CNTs as transport channels break the permeance/selectivity tradeoff exhibited in conventional nanoporous materials (Figure 3d).^[^ ^44^ ^]^

**Figure 3:**
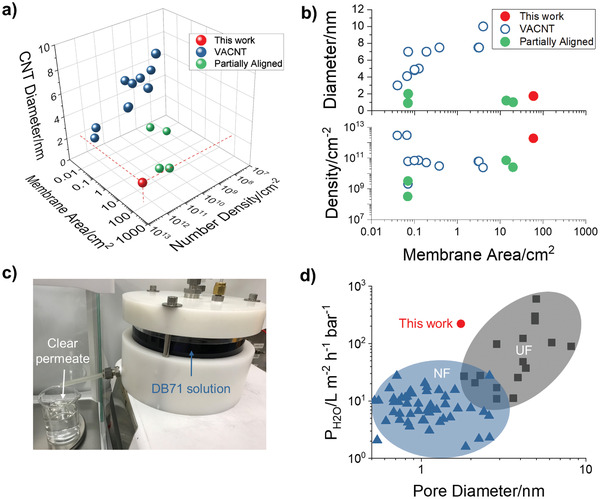
Properties of SWCNT membranes and comparison to literature. a) 3D parameter space comprising CNT diameter, number density, and membrane transport area for reported vertically aligned and partially aligned CNT membranes compared to our large‐area membranes.^[^ ^4^, ^16^, ^17^, ^18^, ^23^, ^33^, ^39^, ^45^, ^46^, ^47^, ^48^, ^49^, ^50^, ^51^, ^52^, ^53^ ^]^ b) 2D projections of CNT number density and tube diameter as a function of membrane transport area. c) Image of 60 cm^2^ membrane rejecting over 99% of the DB71 probe analyte. d) Water permeance reported for commercial nanofiltration (NF, 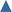) and ultrafiltration (UF, 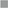) membranes compared to this work (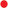). Commercial product information is reported in Tables S6 and S7 in the Supporting Information.

Ultra‐permeable membranes with nanometer‐sized pores are desirable in many nanofiltration and ultrafiltration applications where both pore size and charge often determine selectivity. For example, nanoporous membranes have been investigated for the fractionation and recovery of useful ionic species in waste streams containing pharmaceuticals,^[^ ^54^ ^]^ heavy metals,^[^ ^55^ ^]^ and dyes.^[^ ^56^ ^]^ Waste streams from these processes tend to have high osmotic pressures that impede low cost separations, and selective removal of ionic species could allow for reuse of materials at lower operating pressures.^[^ ^57^, ^58^ ^]^

To understand the applicability for ionic fractionation, we challenged our CNT membranes with single‐component solutions and mixtures of salts and dyes (**Figure** **4**). The rejection characteristics of charged nanoporous membranes, including CNT membranes,^[^ ^35^, ^36^, ^59^ ^]^ are sensitive to the operating conditions including temperature, applied pressure, feed composition, and pH. As shown in Figure 4a, the negatively charged carboxylate species introduced on the CNT tips during membrane etching electrostatically repel monovalent and divalent salts at low ionic strengths. However, the rejection decreases dramatically for both species as the Debye length of the ions approaches the CNT diameter with increasing ionic strength of the solution. The diminished electrostatic repulsion eventually allows greater than 99% of the ions to permeate. Ion valency also contributes to the rejection performance, with the higher valency SO_4_^2−^ anions being repelled more strongly.^[^ ^35^ ^]^ The importance of these electrostatic interactions is underlined by the good agreement between the Donnan model fits with the experimental data (Figure 4a).^[^ ^35^ ^]^ Figure 4c shows the salt rejection as a function of permeate flux during filtration of single‐component solutions at increasing pressure up to 1 bar. The salt rejection initially increases until it reaches a maximum near 50 L m^−2 ^h^−1^ before finally decreasing by almost half. The small rejection increase at low fluxes can be attributed to the increasing water permeance but relatively constant salt permeance with pressure, as seen in many charged polymeric nanofiltration membranes. This effect is likely countered by concentration polarization that begins to dominate at higher fluxes, leading to nearly complete passage of NaCl at 200 L m^−2^ h^−1^ at 1 bar.

**Figure 4:**
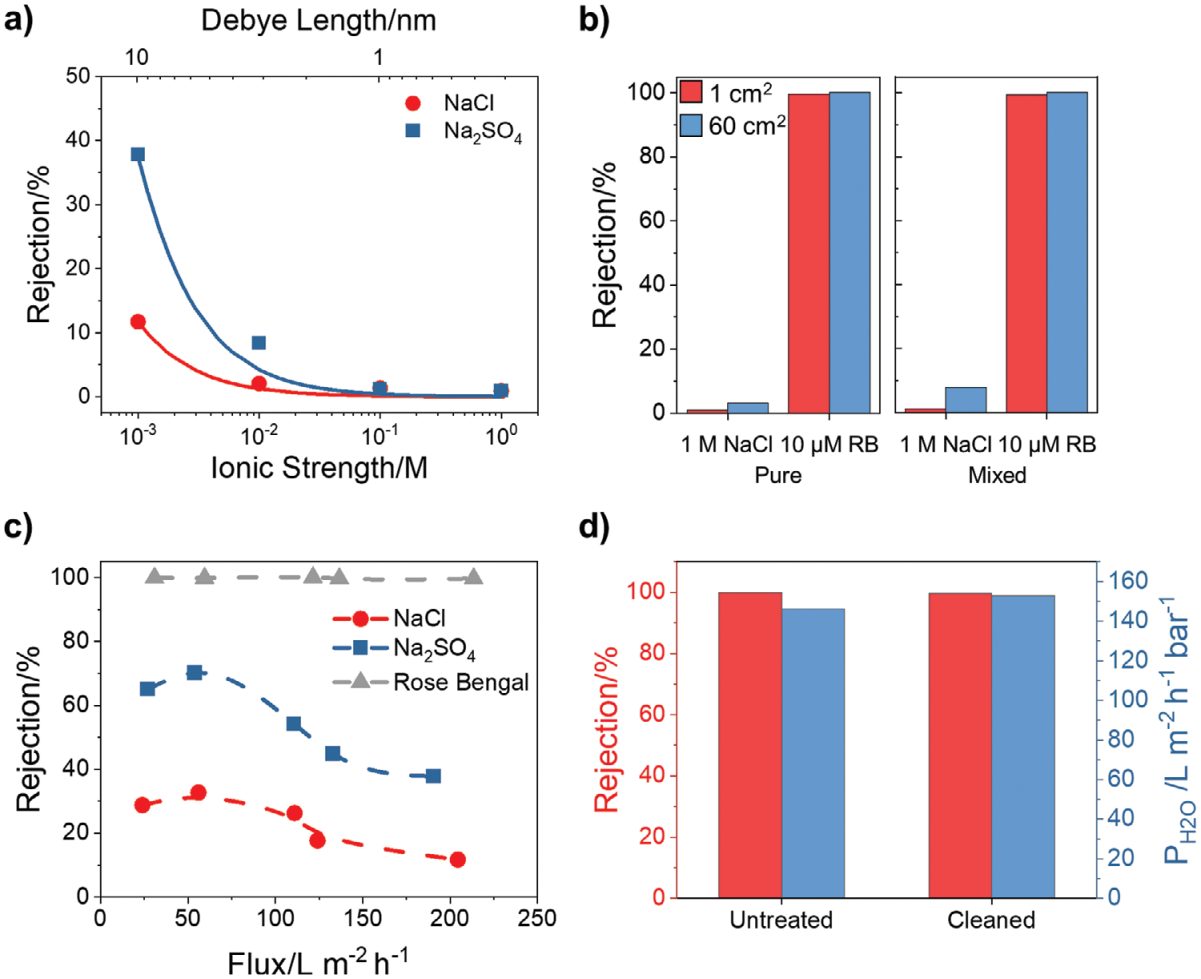
Separation performance of fully open SWCNT membranes. a) NaCl and Na_2_SO_4_ rejection at 1 bar applied pressure for a 1 cm^2^ SWCNT membrane. Lines represent the rejection predicted by the Donnan model. b) Single component (1 m NaCl or 10 × 10^−6^ m Rose Bengal) and mixed feed (10 × 10^−6^ m Rose Bengal in 1 m NaCl) separation performance for 1 and 60 cm^2^ SWCNT membranes. c) Rejection of 1 × 10^−3^ m NaCl, 0.33 × 10^−3^ m Na_2_SO_4_, and 10 × 10^−6^ m Rose Bengal feed solutions at various applied pressures (0.14, 0.28, 0.55, 0.69, and 1 bar) for a 1 cm^2^ SWCNT membrane. Lines used to guide the eye. d) Rose Bengal rejection (left axis) and solution permeance (right axis) for a 1 cm^2^ membrane before and after 2 h exposure to a 2000 ppm bleach cleaning solution.

Intriguing rejection behavior is observed for a representative small dye, Rose Bengal (1.2 × 1.3 × 0.9 nm^3^),^[^ ^60^ ^]^ selected for these proof‐of‐concept dye/salt fractionation experiments because of its high purity. Rose Bengal rejection from low concentration single component solutions remains virtually unchanged even at high fluxes (Figure 4c), thus displaying a much lower sensitivity to concentration polarization than monovalent and divalent salts. Even after soaking the membrane in the feed solution for 72 h, the rejection remains constant, indicating that dye adsorption onto the membrane does not contribute significantly to the rejection results. Negligible dye adsorption was confirmed with independent tests (Figure S1, Supporting Information). Surprisingly, Figure 4b reveals that a greater than 99% Rose Bengal rejection with nearly complete passage of NaCl is maintained when moving from single‐ to multicomponent feeds with high ionic strength (10 × 10^−6^ m Rose Bengal in 1 m NaCl). While a mechanistic description of this phenomenon is beyond the scope of this work, complete dye rejection at high salinities is possibly due to competing effects between charge screening and salt‐induced aggregation of the dye.^[^ ^61^ ^]^ This attractive performance exhibited by 1 cm^2^ membranes is maintained by the wafer‐scale membranes.

Chemical stability is critically important for membrane materials in water treatment since it allows for aggressive cleaning to remove foulants without deterioration of the membrane performance. Both CNTs and parylene N are known to be stable in chemically aggressive solutions, and thus we expected our CNT composite membranes to also display excellent chemical resistance. We investigated the oxidative stability by exposing a CNT membrane to a 2000 ppm bleach solution for 2 h. The Rose Bengal rejection and solution permeance before and after bleach treatment are shown in Figure 4d. As confirmed with other CNT membranes, there is no deterioration in the dye rejection and little change in the permeance.^[^ ^23^ ^]^ This experiment clearly corroborates the excellent stability of the CNT/parylene composites in chemically harsh environments.

In summary, this work demonstrates the fabrication of large‐area (60 cm^2^) CNT membranes with a high density (1.89 × 10^12^ tubes cm^−2^) of 1.7 nm SWCNT pores. By opening all available CNTs to transport, we quantified pressure‐driven gas (290–340×) and water (6100×) flow enhancements with unprecedented accuracy. These ultra‐permeable membranes have water permeances exceeding 200 L m^−2^ h^−1^ bar^−1^, far higher than conventional nano‐ and ultrafiltration membranes with similar pore diameters. We further demonstrated a 60‐fold scale up in membrane transport area compared to previous 1 cm^2^ samples without compromising transport and rejection properties. Our work represents the largest defect‐free VACNT membranes fabricated to date. The reliable production of high‐performance CNT membranes at wafer scale shown here is a first but important step forward for their transition from laboratory toward real world applications. While the employed wafer‐based technology is not suitable for large‐scale manufacturing, recent industrial demonstration of continuous synthesis of vertically aligned SWCNTs^[^ ^62^ ^]^ on metal foils could soon enable membrane scale up to even larger, commercially attractive sizes and reduce the now high production cost to a more competitive level. Owing to their excellent oxidative cleaning resistance, these CNT/parylene composite membranes are promising as highly permeable yet selective membranes for use in chemically demanding applications.

## Conflict of Interest

The authors declare no conflict of interest.

## Supporting information

Supporting Information
