# Supplementary material for: Ultra‐Permeable Single‐Walled Carbon Nanotube Membranes with Exceptional Performance at Scale
Source: Adv Sci (Weinh). 2020 Nov 9;7(24):2001670. doi: 10.1002/advs.202001670 (PMC7740080; doi:10.1002/advs.202001670)
Supplement: Supplementary file 1 — Supporting Information [file ADVS-7-2001670-s001.pdf]

© 2020 Wiley-VCH GmbH

ADVANCED SCIENCE
Open Access

Supporting Information

for *Adv. Sci.,* DOI: 10.1002/advs.202001670

Ultra-Permeable Single-Walled Carbon Nanotube Membranes with Exceptional Performance at Scale

*Melinda L. Jue, Steven F. Buchsbaum, Chiatai Chen, Sei Jin Park, Eric R. Meshot, Kuang Jen J. Wu, and Francesco Fornasiero**

Copyright © 2020 Wiley-VCH GmbH

Supporting Information

Ultra-permeable Single-walled Carbon Nanotube Membranes with Exceptional Performance at Scale

*Melinda L. Jue, Steven F. Buchsbaum, Chiatai Chen, Sei Jin Park, Eric R. Meshot, Kuang Jen J. Wu, Francesco Fornasiero**

Materials

Gases used for CNT synthesis (acetylene, hydrogen, argon) and permeation (helium, nitrogen, argon, and carbon dioxide) were 99+% pure. Hydrochloric acid (37%, ACS grade), isopropyl alcohol (ACS grade), Direct Blue 71 (dye content 50%), Rose Bengal sodium salt (dye content 95%), Eosin Y (dye content ~99%), Erythrosin B (dye content ≥ 95%), Thymolphthalein (dye content 95%), *m*-Cresol Purple (dye content 90%), and sodium chloride (≥ 99.5%), were purchased from Sigma Aldrich. Sodium sulfate (99.2%) was purchased from Fisher Scientific. Aqueous solution of polyethylene glycol (PEG) coated gold nanoparticle (5 nm, 0.05 mg mL^{-1}) was purchased from nanoComposix. Aqueous solutions of PVP capped spherical gold nanoparticles (1.8, 2.2, 3, 4, and 5 nm, 0.06 mg mL^{-1}) and CTAB capped spherical gold nanoparticles (1.8, 2.2, 3, 4, and 5 nm, 0.06 mg mL^{-1}) were purchased from Nanopartz Inc. Concentrated bleach (Germicidal Bleach, Clorox®) was used in the cleaning solution. All chemicals were used as received.

Carbon Nanotube Synthesis

Vertically aligned carbon nanotubes were grown on 100 mm Si (100) wafers that were coated with a Fe/Mo/Al_2O_3 (5.5/0.5/300 Å) catalyst stack by electron-beam evaporation without breaking vacuum between layers (base pressure ≤ 1.6 x 10^{-6} mbar). The thicknesses of the catalyst layers were recorded in situ by a quartz crystal balance. CNT synthesis was performed by low pressure chemical vapor deposition in a cold-wall furnace (AIXTRON® Black Magic Pro 6 in.) with acetylene as the hydrocarbon feedstock, as described in detail elsewhere.[1]

The average CNT number density N (cm^{-2}) of produced forests was calculated using the weight gain method with Equation (S1)

$$N = \frac{\rho SSA_g}{\pi d} \qquad \text{(S1)}$$

where ρ is the volumetric mass density, SSA_g is the specific surface area of graphene (1315 m^2 g^{-1}), and d is the mean SWCNT diameter from TEM measurements.[2] This method has been shown to agree well with other techniques such as X-ray attenuation.[3] The volumetric mass density was obtained from the mass gain of the catalyst-coated silicon wafer after CNT growth and calculated according to Equation (S2)

$$\rho = \frac{m_{CNT}}{h_{CNT} A_{CNT}} \qquad \text{(S2)}$$

where m_{CNT} is the mass of the CNT forest, h_{CNT} is the height of the CNT forest, and A_{CNT} is the CNT growth area. Approximately 30 mg of VACNTs were grown on each wafer and measured with 0.1 mg precision. Possible error on m_{CNT} quantification due to amorphous carbon content amounts to < 5%, as revealed by previous TGA measurements.[1] The CNT forest height (~40 µm) was measured by optical microscopy as the difference between the z-location of the water surface (without CNTs) and the top of the CNT forest when each plane was in focus (0.1 µm

resolution). A weighted average of 5 height measurements at different locations along the wafer meridian was used for calculations.

Carbon Nanotube and Composite Membrane Imaging

The SWCNT diameter distribution was determined using high-resolution transmission electron microscopy (HRTEM, 2100-F field-emission analytical TEM, JEOL) from a sample of more than 100 CNTs (0.01 nm resolution). CNT samples were harvested from pristine forests prior to membrane fabrication, sonicated in ethanol, and drop cast onto Formvar coated Cu TEM grids. To extract the CNT diameter, defined here as the distance between the CNT wall centers, collected TEM images were analyzed using a custom MATLAB script.[1] Note that the inner pore diameter is actually 0.34 nm smaller (~1.4 ± 0.7 nm) than the reported average CNT diameter defined above (1.7 nm ± 0.7).

Cross-sectional scanning electron microscopy images of the composite membranes were taken by an Apreo (Thermo Scientific) SEM at 3 kV accelerating voltage and spot size 6. ImageJ software was used to measure the membrane thickness. CNT composites employed in this work were 36 ± 4 µm thick.

Carbon Nanotube Membrane Fabrication

The composite membranes were created by vapor deposition of poly-para-xylylene (parylene N) within vertically aligned CNT forests as described in detail elsewhere[3-5]. The polymer coating fills the interstitial gaps between the tubes and leaves a small excess layer on the top of the membrane surface. Excess parylene N and the CNT caps were removed using an inductive super magnetron (ISM) generated oxygen plasma (NE-550EXa, ULVAC). The antenna power was set

to 200 W, bias power to 20 or 25 W, chiller to 0 °C, pressure to 0.5 Pa, and O_2 flow rate to 99 sccm. To estimate the etch rate under these etch conditions, the average thickness of a parylene N layer on a silicon wafer was measured after multiple etch steps using non-contact spectro-reflectometry (TOHO 3100, NanoSpec). Data were fit to a Cauchy model with both thickness and index of refraction as fitting parameters. The wafers were submerged in hydrochloric acid for several hours to remove the catalyst particles and separate the composite from the silicon wafer by dissolving the intermediate alumina thin film.

The free-standing composite membranes were glued (silicone glue, 734 Flowable Adhesive Sealant, Dow Corning) to 127 µm thick annular polyimide films (Kapton® HN, DuPont) that acted as support frames. Large membranes utilized the entire composite formed from 100 mm diameter CNT forests, while small membranes were cut from larger membranes using a hammer-driven hole punch.

Control Membranes with Blocked CNTs

Control membranes with internally clogged CNTs were fabricated following the same protocol described above, with the only difference being that the CNT tips of the forests were removed via a short air plasma etch after growth (5 min, 30 W RF, Harrick PDC-001). Thus, during the following parylene N deposition step, both the inter-tube spaces and the inner volume of the uncapped CNT tubes were filled by polymer. As detailed in our separate work,[4] these control membranes with intentionally clogged CNT channels did not transport fluids even after extensive etching beyond the limit required to open membranes in this study. Together with the detailed transport and rejection tests, data collected from these control membranes strongly suggests that all recorded flow in our standard CNT membranes is through the CNTs only.

Gas Permeation

The gas transport rates through the composite membranes were measured in custom-built dead-end permeation cells at ambient conditions (21 °C). The upstream of the membrane was flushed several times and the system was allowed to come to equilibrium before measurement. The feed was pressurized, and the downstream flow rate was measured using mass flow meters.

The obtained permeation data for each gas was used to calculate the enhancement factor, defined as the relative ratio of the measured CNT membrane permeance over the Knudsen diffusion prediction. The Knudsen permeance P_k is given by Equation (S3)

$$P_k = \frac{\varepsilon d}{3\tau L}\left(\frac{8}{\pi MRT}\right)^{1/2} \tag{S3}$$

where ε is the porosity, d is the CNT diameter, τ is the tortuosity, R is the universal gas constant, T is temperature, L is the membrane thickness, and M is the molar mass of the gas. In these calculations, we assumed a tortuosity of 1.25 based on our previous results.[3]

For estimating gas and liquid flow enhancements, we assumed that all CNTs in the membranes are open to fluid flow at the gas permeance plateau. While we cannot experimentally validate that 100% of the CNTs are conducting fluids via other methods, we believe that our assumption is reasonable. Indeed, since the CNTs are capped during polymer infiltration, significant pore clogging by the vapor-deposited parylene N is unlikely. SWCNTs cannot form bamboo structures and do not display the tendency to be internally blocked by catalyst particles. Finally, the Fe/Mo nanoparticles at the nanotube base are dissolved in the hydrochloric acid soaking step used for our membrane delamination. Thus, any overestimate of the number of open pores (and resulting underestimate of flow enhancements) is expected to be much more modest than in other experimental studies. As we explained in the main text and contrary to our work,

published literature rarely demonstrates achieving a transport rate plateau before assuming that all the CNTs are open for transport and/or uses unreliable methods to extract the number of open pores.

Liquid Permeation

The liquid permeation experiments were conducted in the same custom-built dead-end permeation cells. The membranes were first soaked in isopropyl alcohol for 3 minutes before being rinsed thoroughly with deionized water to ensure wetting of all the hydrophobic CNT pores. The feed was stirred using a magnetic stirrer suspended above the membrane and pressurized using nitrogen gas. The permeate weight was collected in a glass vial and automatically recorded on a digital balance (Adventurer AX, Ohaus).

The pure water flux was measured after the gas permeation experiments, but before any dye or salt experiments to avoid potential pore blocking or fouling. The enhancement factor for liquid permeation was defined as the relative ratio of the measured per-CNT flow rate (membrane flow rate normalized by the CNT number density) over the estimated transport rate assuming no-slip Hagen-Poiseuille flow

$$Q_{hp} = \frac{\pi\left(\frac{d}{2}\right)^4 \Delta p}{8\mu L\tau} \tag{S4}$$

where d is the average CNT diameter (1.7 ± 0.7 nm), Δp is the pressure drop across the membrane ($2.677 \times 10^{-1} \pm 3 \times 10^{-4}$ bar), μ is the dynamic viscosity of water at the experimental temperature of 21 °C (9.76×10^{-4} Pa s), L is the membrane thickness (36 ± 4 μm), and τ is the tortuosity (1.25).

For liquid permeation experiments, ~80 mL and ~500 mL of feed solution were used with small and large area membranes, respectively. The feed solutions consisted of Direct Blue 71 (10

µM), 5 nm diameter PEG-coated gold nanoparticles (0.05 mg mL^{-1}), sodium chloride (1, 10, 100, and 1000 mM), sodium sulfate (0.33, 3.3, 33, and 333 mM), and Rose Bengal sodium salt (10 µM) in deionized water. Rose Bengal (95% dye content) was selected in the dye/salt fractionation study to avoid complications resulting from low purity dyes typically used with textiles (~30% dye content). For easier rejection data interpretation, the Rose Bengal concentration was kept below the threshold for aggregation in aqueous solution.[6] The mixed dye and salt feed consisted of Rose Bengal sodium salt (10 µM) with sodium chloride (1 M) in deionized water. The membranes were rinsed thoroughly with deionized water before each test and stored in water when not in use.

Simulated bleach cleaning was performed by soaking the membranes in a 2000 ppm bleach solution for 2 hours. The cleaned membranes were washed thoroughly with deionized water before testing.

Rejection Measurements

To exclude the presence of membrane defects in both small and large area samples, we performed pressure-driven filtration experiments with two test solutions: aqueous 10 µM Direct Blue 71 and 5 nm PEG-coated gold nanoparticles (0.05 mg mL^{-1}). Small area membranes were also subjected to additional rejection tests with an expanded set of neutral PVP-coated gold nanoparticles (1.8, 2.2, 3, 4, and 5 nm, 0.06 mg mL^{-1}), positively charged CTAB-coated gold nanoparticles (1.8, 2.2, 3, 4, and 5 nm, 0.06 mg mL^{-1}), and high purity dyes (10 µM Eosin Y, 5 µM Erythrosin B, 30 µM Thymolphthalein, 10 µM *m*-Cresol Purple). The membrane rejection R of these analytes and Rose Bengal was quantified from the feed (A_f) and permeate (A_p)

absorbances measured by UV-vis spectroscopy (Cary 100 UV-visible spectrophotometer, Agilent; scan spacing: 1 nm; scan range: 200-800 nm) according to Equation (S5):

$$R = \left(1 - \frac{A_p}{A_f}\right) \times 100 \tag{S5}$$

For NaCl and Na_2SO_4 rejection quantification, the absorbances in Equation (4) were replaced by the permeate and feed solution conductivities measured at room temperature (21 °C) with a 712 Conductometer (Metrohm AG).

Sorption Measurements

Sorption experiments were performed using Rose Bengal (1×10^{-5} M) and 5 nm PEG-coated gold nanoparticle (0.05 mg mL^{-1}) solutions. 1 cm^2 CNT composite membrane samples were soaked in 20 mL of each feed for 72 hours. This contact time is nearly two orders of magnitude longer than a typical rejection test. The UV-vis spectra along with images of the solution vials before and after membrane soaking are shown in Figure S1. The solution concentrations remained unchanged or increased only slightly after exposure to the membrane, suggesting that minor water loss due to evaporation around the seal of the vial is more significant than any dye adsorption onto the CNT membrane itself.

Furthermore, the membrane rejections before and after sorption tests were measured. Membranes showed > 99% rejection for both the Rose Bengal and 5 nm PEG-coated gold nanoparticle solutions even after soaking in solution for 72 hours.

These results demonstrate that dye/particle adsorption on the membrane is negligible and cannot account for the complete dye/particle rejection observed during filtration experiments (Figure 2f).

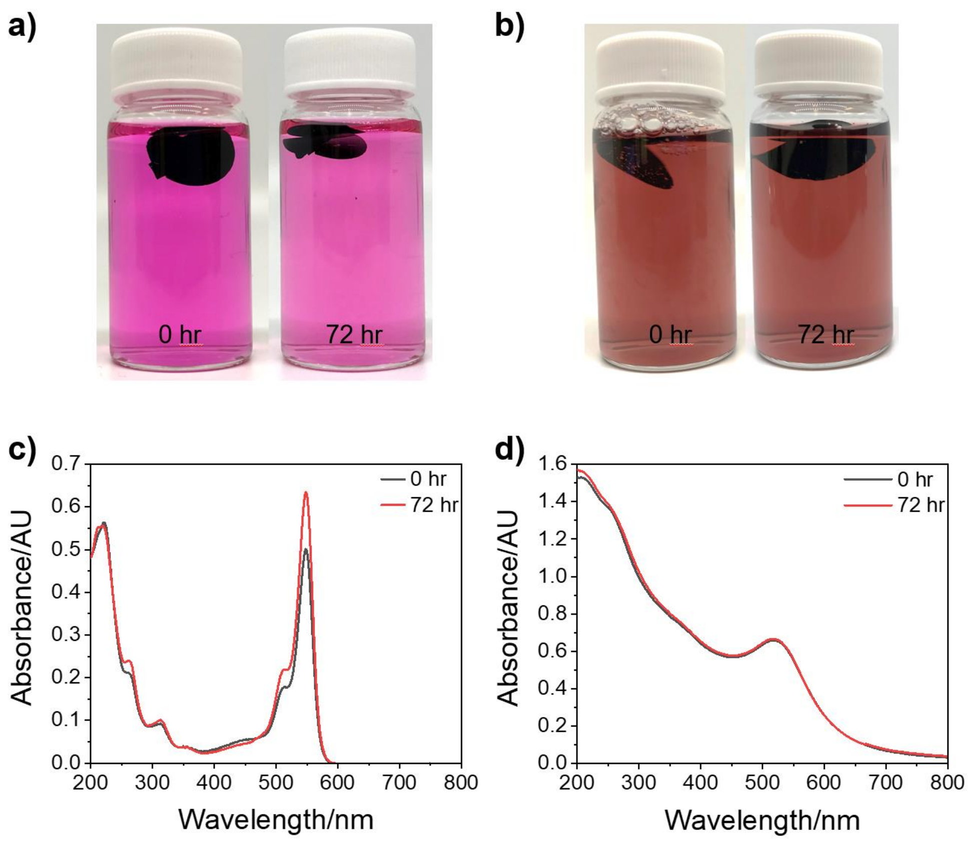

Figure S1. Dye and nanoparticle adsorption data. Pictures of a) 1 x 10^{-5} M Rose Bengal solution and b) 0.05 mg mL^{-1} 5 nm PEG-coated gold nanoparticle solution at 0 and 72 hours of exposure to a 1 cm^2 CNT composite membrane. Corresponding UV-vis spectra at 0 and 72 hours for c) 1 x 10^{-5} M Rose Bengal solution and d) 0.05 mg mL^{-1} 5 nm PEG-coated gold nanoparticle solution.

Donnan Exclusion Modeling

The Donnan model was used to predict the rejection behavior of ions through a charged CNT membrane according to Equation (S6)

$$R = 1 - \frac{c_i^m}{c_i} = 1 - \left(\frac{|z_i| c_i}{|z_i| c_i^m + c_x^m}\right)^{|z_i/z_j|} \quad \text{(S6)}$$

where c_i^m is the anion concentration in the membrane, c_i is the anion concentration in solution, c_x^m is the membrane charge concentration, and z_i and z_j are the anion and cation charge,

respectively.[7] The membrane charge concentration was fit to the experimental salt rejection data at the lowest salt concentration (1 mM NaCl and 0.33 mM Na_2SO_4) and then kept constant to estimate the rejection at higher concentrations.

Raman Spectroscopy

Micro-Raman spectroscopy (inVia™ Qontor® confocal Raman microscope, Renishaw) with an excitation wavelength of λ = 633 nm and grating of 1200 lines mm^{-1} was used to quantify the quality of the CNTs by determining the G-band (~1590 cm^{-1}) to D-band (~1310 cm^{-1}) ratio. The radial breathing mode peak intensities were normalized to the D-band peak area. Raman spectra were measured at the center and edge of the CNT wafer before coating with parylene N. High G/D band values (6 and 6.8 at the center and edge, respectively) indicate good structural quality and spatial uniformity of the CNTs across large area.

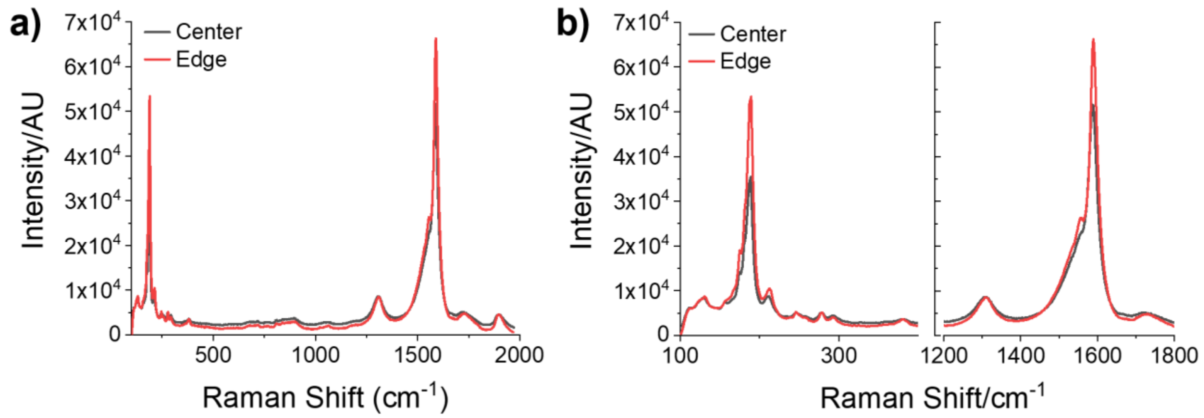

Figure S2. a) Raman spectra for wafer-scale CNT forests at the center and edge. b) Spectral regions corresponding to the RBM peaks (left) and G- and D-bands (right). Spectra are normalized to the D-band.

Tabulated Data

Table S1. Direct Blue 71 and 5 nm PEG-coated gold nanoparticle solution rejection data at 0.28 bar applied pressure for the 1 cm^2 membrane in Figure 1f.

Feed Solution	Rejection (%)
Direct Blue 71	99.4 ± 0.3
PEG-coated Gold Nanoparticles	99.8

Table S2. Experimentally measured average enhancement factors for gas flow through CNT membranes shown in Figure 2b.

Reference	CNT Diameter (nm)	Helium	Nitrogen	Carbon Dioxide	Argon
This work	1.7 ± 0.7	290 ± 20	320 ± 50	320 ± 30	340 ± 50
Ge[8]	7.7	-	56	43	40
Zhang[9]	7	69	61	58	64
Zhang[10]	7	37	36	39	35
Holt[11]	1.6	70	73	76	76
McGinnis[12]	1	70	30	-	-

Table S3. Experimentally measured average enhancement factors for pure water flow through CNT membranes shown in Figure 2d.

Mean CNT Diameter (nm)	Water Enhancement Factor	Reference
1.7 ± 0.7	6100 ± 500	This work
1.6	2220	Holt[11]
3.3	214	Bui[3]
3.3	379	Kim[13]
7	60000	Majumder[14]
4.8	69000	Baek[15]
0.81	882	Qin[16]
0.87	662	Qin[16]
0.98	354	Qin[16]
1.1	580	Qin[16]
1.42	103	Qin[16]
1.52	59	Qin[16]
1.59	51	Qin[16]

Table S4. Simulation results of the enhancement factor for pure water through CNTs shown in Figure 2d.

Mean CNT Diameter (nm)	Water Enhancement Factor	Reference
1.62	870	Kannam[17]
1.9	680	Kannam[17]
2.16	525	Kannam[17]
2.72	350	Kannam[17]
3.26	260	Kannam[17]
3.8	210	Kannam[17]
4.34	180	Kannam[17]
4.88	160	Kannam[17]
6.5	90	Kannam[17]
1	4020[a]	Falk[18]
2	878[a]	Falk[18]
3	441[a]	Falk[18]
4	282[a]	Falk[18]
6	155[a]	Falk[18]
7	125[a]	Falk[18]
8	104[a]	Falk[18]
10	77[a]	Falk[18]
2	253	Walther[19]
2	350	Borg[20]
3	184	Borg[20]
4	132	Borg[20]
6	86	Borg[20]
8	64	Borg[20]
10	51	Borg[20]
1.5	717[a]	Thomas[21]
2	297[a]	Thomas[21]
3	116[a]	Thomas[21]
4	72[a]	Thomas[21]
6	43[a]	Thomas[21]
8	32[a]	Thomas[21]
10	25[a]	Thomas[21]

a) Calculated from slip length data using $E = \left(1 + 8\frac{L_s}{d}\right)$ where E is the enhancement factor, L_s is the slip length, and d is the CNT diameter.

Table S5. CNT composite membrane properties shown in Figure 3a and b.

Type	Mean CNT Diameter (nm)	Membrane Area (cm^2)	CNT Number Density (cm^{-2})	Reference
Vertically Aligned	1.7 ± 0.7	59.6	1.89×10^{12}	This work
Vertically Aligned	3	0.04	2.9×10^{12}	Yu[22]
Vertically Aligned	4.1	0.067	3×10^{12}	Lee[23]
Vertically Aligned	4.8	0.1	6.8×10^{10}	Baek[15]
Vertically Aligned	4.87	0.1	6.8×10^{10}	Park[24]
Vertically Aligned	5	0.126	7×10^{10}	Shadmehr[25]
Vertically Aligned	7	0.19	5×10^{10}	Majumder[26]
Vertically Aligned	7	0.071	6×10^{10}[a]	Sun[27]
Vertically Aligned	7	0.071	2.03×10^{9}[a]	Zhang[10]
Vertically Aligned	7	0.071	2.08×10^{9}[a]	Zhang[9]
Vertically Aligned	7.5	3.1	6×10^{10}	Hinds[28]
Vertically Aligned	7.5	3.2	6×10^{10}	Nednoor[29]
Vertically Aligned	7.5	0.38	3×10^{10}	Pilgrim[30]
Vertically Aligned	10	4	2.4×10^{10}	Du[31]
Partially Aligned	0.9	0.07	3.14×10^{8}[a]	Wu[32]
Partially Aligned	1	19.9	2.5×10^{10}	McGinnis[12]
Partially Aligned	1.2	13.9	7×10^{10}	Kim[33]
Partially Aligned	2	0.071	3.2×10^{9}[a]	Sun[27]

a) Calculated from reported membrane properties.

Table S6. Reported pure water permeance for commercially available nanofiltration membranes in Figure 3d. Listed pore diameters are typically obtained from molecular weight cut-off measurements.

Membrane	Material	Pore Diameter (nm)	Permeance (L/m^2hbar)	Reference
NF 2A	Polyamide	0.52	10.1	Lin[34]
HC-50	Polyamide	0.54	2.08	Garcia-Aleman[35]
NF-90	Polyamide	0.55	10.2	Hilal[36]
NF-90	Polyamide	0.68	6.4	Nghiem[37]
NF-90	Polyamide	0.73	9.6	Bargeman[38]
NF-90	Polyamide	0.76	11.3	López-Muñoz[39]
NF-90	Polyamide	1.36	4.90	Nicolini[40]
N30F	Polyethersulfone	0.61	4.45	Hilal[36]
NF 6	Polyamide	0.64	16.7	Lin[34]
NF-70	Polyamide	0.68	9.53	Van der Bruggen[41]
NF-270	Polyamide	0.68	11	Braeken[42]
NF-270	Polyamide	0.71	27.5	Hilal[36]
NF-270	Polyamide	0.8	11.5	Bargeman[38]
NF-270	Polyamide	0.84	13.5	Nghiem[37]
NF-270	Polyamide	0.88	13.5	López-Muñoz[39]
NF-270	Polyamide	1.05	20.5	Werber[43]
NF-270	Polyamide	1.08	14.8	Lin[44]
TS-80	Polyamide	0.74	6	Bargeman[38]
TS-82	-	0.74	4.7	Bargeman[38]
HL	-	0.82	9.01	Hu[45]
HL	-	0.82	12	Pan[46]
Desal-HL-51	Polyamide	0.96	9	Braeken[42]
Desal 5DK	Polyamide	0.83	4.2	Bargeman[38]
Desal 5DK	-	1.5	5.17	Pan[46]
NTR-7250	Polypiperazine amide	0.85	3.6	Bargeman[38]
NFT50	Polypiperazine amide	0.86	5.9	Teixeira[47]
UTC-20	Polypiperazine amide	0.86	15	Braeken[42]
NF70	Polyamide	0.96	6.72	Lin[44]
NF-45	Polyamide	1.02	4.57	Pan[46]
NF-45	Polyamide	1.04	4.56	Garcia-Aleman[35]
UTC-20	Polyamide	1.08	17.4	Van der Bruggen[41]
XN-45	Polyamide	1.09	6.7	Bargeman[38]
HN-7450	Polyethersulfone	1.12	3.06	Garcia-Aleman[48]

HN-7450	Polyethersulfone	2.24	2.19	Garcia-Aleman[35]
NF 250	Polysulfone	1.12	6.30	Kotrappanavar[49]
NF-2	-	1.14	8.53	Pan[46]
DL	-	1.16	7.63	Hu[45]
DL	-	1.16	7.29	Pan[46]
NF 300	Polysulfone	1.24	7.05	Kotrappanavar[49]
TFC-SR2	Polyamide	1.28	15.4	Nghiem[37]
NTR-7470	Polysulfone	1.3	4.3	Bargeman[38]
NTR-7450	Sulf.[a] Polyethersulfone	1.4	12	Braeken[42]
NTR-7450	Sulf.[a] Polyethersulfone	1.6	15.7	Van der Bruggen[41]
NTR-7450	Sulf.[a] Polyethersulfone	1.67	7.4	Bargeman[38]
SR-1	-	1.4	8.81	Hu[45]
NP030	Polyethersulfone	1.86	1.60	Nicolini[40]
NF-PES-010	Polyethersulfone	2	7.5	Braeken[42]
Desal G-20	-	2.4	6.5	Afonso[50]
NP010	Polyethersulfone	2.58	5.50	Nicolini[40]
Desal G-10	-	2.6	2.3	Afonso[50]
NTR-7410	Sulf.[a] Polyethersulfone	2.6	27.6	Bargeman[38]

a) Sulf. = sulfonated.

Table S7. Reported pure water permeance for commercially available ultrafiltration membranes in Figure 3d. Listed pore diameters are obtained from molecular weight cut-off measurements.

Membrane	Material	Pore Diameter (nm)	Permeance (L/m^{2}hbar)	Reference
UH004	Polyethersulfone	1.82	27	Lin[51]
UH004	Polyethersulfone	2.12[a]	20.7	Penha[52]
UP005	Polyethersulfone	2.28[a]	27.8	Penha[52]
UF PES-10 kDa	Polyethersulfone	2.87[a]	10.9	Zulaikha[53]
UP010	Polyethersulfone	2.87[a]	97.8	Penha[52]
10 PES	Polyethersulfone	2.87[a]	17.1	de Souza Araki[54]
UF-commercial	-	3.62[a]	11.1	Celik[55]
UP5	Polyethersulfone	3.86	25.6	Jiang[56]
30 PVDF	Polyvinylidene fluoride	4.14[a]	123	de Souza Araki[54]
UF	Polyethersulfone	4.14[a]	48.3	Maruf[57]
GR82PP	Polyethersulfone	4.32	37.3	Jiang[56]
UH050	Polyethersulfone	4.91[a]	250	Penha[52]
50 PVDF	Polyvinylidene fluoride	4.91[a]	300	de Souza Araki[54]
100 kD	Regenerated cellulose	5	597	Singh[58]
UF PVDF-100 kDa	Polyvinylidene fluoride	6.19[a]	104	Zulaikha[53]
UP10	Polyethersulfone	8.14	89.3	Jiang[56]

a) Pore diameter estimated from the molecular weight cutoff.[59]

References

[1] E. R. Meshot, S. J. Park, S. F. Buchsbaum, M. L. Jue, T. R. Kuykendall, E. Schaible, L. B. Bayu Aji, S. O. Kucheyev, K. J. J. Wu, F. Fornasiero, *Carbon* **2020**, *159*, 236.

[2] S. Esconjauregui, R. Xie, M. Fouquet, R. Cartwright, D. Hardeman, J. Yang, J. Robertson, *J. Appl. Phys.* **2013**, *113*, 144309.

[3] N. Bui, E. R. Meshot, S. Kim, J. Peña, P. W. Gibson, K. J. Wu, F. Fornasiero, *Adv. Mater.* **2016**, *28*, 5871.

[4] S. F. Buchsbaum, M. L. Jue, A. Sawvel, C. Chen, E. R. Meshot, S. J. Park, M. Wood, K. J. Wu, C. Bilodeau, F. Aydin, A. Pham, E. Lau, F. Fornasiero, *Adv. Sci.* **2020**, under review.

[5] Y. Li, C. Chen, S. F. Buchsbaum, E. Meshot, M. B. Herbert, R. Zhu, O. Kulikov, B. R. McDonald, N. Bui, M. L. Jue, S. J. Park, C. Valdez, S. Hok, K. J. Wu, T. M. Swager, F. Fornasiero, *Adv. Funct. Mater.* **2020**, *30*, 2000258.

[6] D. Xu, D. Neckers, *J. Photochem. Photobiol. A: Chem.* **1987**, *40*, 361.

[7] F. Fornasiero, H. G. Park, J. K. Holt, M. Stadermann, C. P. Grigoropoulos, A. Noy, O. Bakajin, *Proc. Natl. Acad. Sci.* **2008**, *105*, 17250.

[8] L. Ge, L. Wang, A. Du, M. Hou, V. Rudolph, Z. Zhu, *RSC Adv.* **2012**, *2*, 5329.

[9] L. Zhang, J. Yang, X. Wang, B. Zhao, G. Zheng, *Nanoscale Res. Lett.* **2014**, *9*, 448.

[10] L. Zhang, B. Zhao, X. Wang, Y. Liang, H. Qiu, G. Zheng, J. Yang, *Carbon* **2014**, *66*, 11.

[11] J. K. Holt, H. G. Park, Y. Wang, M. Stadermann, A. B. Artyukhin, C. P. Grigoropoulos, A. Noy, O. Bakajin, *Science* **2006**, *312*, 1034.

[12] R. L. McGinnis, K. Reimund, J. Ren, L. Xia, M. R. Chowdhury, X. Sun, M. Abril, J. D. Moon, M. M. Merrick, J. Park, K. A. Stevens, J. R. McCutcheon, B. D. Freeman, *Sci. Adv.* **2018**, *4*.

[13] S. Kim, F. Fornasiero, H. G. Park, J. B. In, E. Meshot, G. Giraldo, M. Stadermann, M. Fireman, J. Shan, C. P. Grigoropoulos, O. Bakajin, *J. Membr. Sci.* **2014**, *460*, 91.

[14] M. Majumder, N. Chopra, B. J. Hinds, *ACS Nano* **2011**, *5*, 3867.

[15] Y. Baek, C. Kim, D. K. Seo, T. Kim, J. S. Lee, Y. H. Kim, K. H. Ahn, S. S. Bae, S. C. Lee, J. Lim, K. Lee, J. Yoon, *J. Membr. Sci.* **2014**, *460*, 171.

[16] X. Qin, Q. Yuan, Y. Zhao, S. Xie, Z. Liu, *Nano Lett.* **2011**, *11*, 2173.

[17] S. K. Kannam, B. D. Todd, J. S. Hansen, P. J. Daivis, *J. Chem. Phys.* **2013**, *138*, 094701.

[18] K. Falk, F. Sedlmeier, L. Joly, R. R. Netz, L. Bocquet, *Nano Lett.* **2010**, *10*, 4067.

[19] J. H. Walther, K. Ritos, E. R. Cruz-Chu, C. M. Megaridis, P. Koumoutsakos, *Nano Lett.* **2013**, *13*, 1910.

[20] M. K. Borg, D. A. Lockerby, K. Ritos, J. M. Reese, *J. Membr. Sci.* **2018**.

[21] J. A. Thomas, A. J. H. McGaughey, *Nano Lett.* **2008**, *8*, 2788.

[22] M. Yu, H. H. Funke, J. L. Falconer, R. D. Noble, *Nano Lett.* **2009**, *9*, 225.

[23] K.-J. Lee, H.-D. Park, *J. Membr. Sci.* **2016**, *501*, 144.

[24] S.-M. Park, J. Jung, S. Lee, Y. Baek, J. Yoon, D. K. Seo, Y. H. Kim, *Desalination* **2014**, *343*, 180.

[25] S. Shadmehr, M. Coleman, B. Liu, J. Liu, X. Tang, *RSC Adv.* **2017**, *7*, 611.

[26] M. Majumder, N. Chopra, R. Andrews, B. J. Hinds, *Nature* **2005**, *438*, 44.

[27] X. Sun, X. Su, J. Wu, B. J. Hinds, *Langmuir* **2011**, *27*, 3150.

[28] B. J. Hinds, N. Chopra, T. Rantell, R. Andrews, V. Gavalas, L. G. Bachas, *Science* **2004**, *303*, 62.

[29] P. Nednoor, N. Chopra, V. Gavalas, L. G. Bachas, B. J. Hinds, *Chem. Mater.* **2005**, *17*, 3595.

[30] G. A. Pilgrim, J. W. Leadbetter, F. Qiu, A. J. Siitonen, S. M. Pilgrim, T. D. Krauss, *Nano Lett.* **2014**, *14*, 1728.

[31] F. Du, L. Qu, Z. Xia, L. Feng, L. Dai, *Langmuir* **2011**, *27*, 8437.

[32] J. Wu, K. Gerstandt, H. Zhang, J. Liu, B. J. Hinds, *Nat. Nanotechnol.* **2012**, *7*, 133.

[33] S. Kim, J. R. Jinschek, H. Chen, D. S. Sholl, E. Marand, *Nano Lett.* **2007**, *7*, 2806.

[34] J. Lin, C. Y. Tang, C. Huang, Y. P. Tang, W. Ye, J. Li, J. Shen, R. Van den Broeck, J. Van Impe, A. Volodin, C. Van Haesendonck, A. Sotto, P. Luis, B. Van der Bruggen, *J. Membr. Sci.* **2016**, *501*, 1.

[35] J. Garcia-Aleman, J. M. Dickson, *J. Membr. Sci.* **2004**, *235*, 1.

[36] N. Hilal, H. Al-Zoubi, A. W. Mohammad, N. A. Darwish, *Desalination* **2005**, *184*, 315.

[37] L. D. Nghiem, S. Hawkes, *Sep. Purif. Technol.* **2007**, *57*, 176.

[38] G. Bargeman, J. B. Westerink, C. F. H. Manuhutu, A. t. Kate, *J. Membr. Sci.* **2015**, *485*, 112.

[39] M. J. López-Muñoz, A. Sotto, J. M. Arsuaga, B. Van der Bruggen, *Sep. Purif. Technol.* **2009**, *66*, 194.

[40] J. V. Nicolini, C. P. Borges, H. C. Ferraz, *Sep. Purif. Technol.* **2016**, *171*, 238.

[41] B. Van der Bruggen, B. Daems, D. Wilms, C. Vandecasteele, *Sep. Purif. Technol.* **2001**, *22-23*, 519.

[42] L. Braeken, B. Bettens, K. Boussu, P. Van der Meeren, J. Cocquyt, J. Vermant, B. Van der Bruggen, *J. Membr. Sci.* **2006**, *279*, 311.

[43] J. R. Werber, C. J. Porter, M. Elimelech, *Environ. Sci. Technol.* **2018**, *52*, 10737.

[44] Y.-L. Lin, P.-C. Chiang, E. E. Chang, *J. Hazard. Mater.* **2007**, *146*, 20.

[45] K. Hu, J. M. Dickson, *J. Membr. Sci.* **2006**, *279*, 529.

[46] B. Pan, P. Yan, L. Zhu, X. Li, *Desalination* **2013**, *317*, 127.

[47] M. R. Teixeira, M. J. Rosa, M. Nyström, *J. Membr. Sci.* **2005**, *265*, 160.

[48] J. Garcia-Aleman, J. M. Dickson, *J. Membr. Sci.* **2004**, *239*, 163.

[49] N. S. Kotrappanavar, A. A. Hussain, M. E. E. Abashar, I. S. Al-Mutaz, T. M. Aminabhavi, M. N. Nadagouda, *Desalination* **2011**, *280*, 174.

[50] M. D. Afonso, G. Hagmeyer, R. Gimbel, *Sep. Purif. Technol.* **2001**, *22-23*, 529.

[51] J. Lin, W. Ye, M.-C. Baltaru, Y. P. Tang, N. J. Bernstein, P. Gao, S. Balta, M. Vlad, A. Volodin, A. Sotto, P. Luis, A. L. Zydney, B. Van der Bruggen, *J. Membr. Sci.* **2016**, *514*, 217.

[52] F. M. Penha, K. Rezzadori, M. C. Proner, V. Zanatta, G. Zin, D. W. Tondo, J. Vladimir de Oliveira, J. C. C. Petrus, M. Di Luccio, *Eur. Polym. J.* **2015**, *66*, 492.

[53] S. Zulaikha, W. J. Lau, A. F. Ismail, J. Jaafar, *Journal of Water Process Engineering* **2014**, *2*, 58.

[54] M. de Souza Araki, C. de Morais Coutinho, L. A. G. Gonçalves, L. A. Viotto, *Sep. Purif. Technol.* **2010**, *71*, 13.

[55] E. Celik, L. Liu, H. Choi, *Water Res.* **2011**, *45*, 5287.

[56] M. Jiang, K. Ye, J. Deng, J. Lin, W. Ye, S. Zhao, B. Van der Bruggen, *Environ. Sci. Technol.* **2018**, *52*, 10698.

[57] S. H. Maruf, L. Wang, A. R. Greenberg, J. Pellegrino, Y. Ding, *J. Membr. Sci.* **2013**, *428*, 598.

[58] N. Singh, Z. Chen, N. Tomer, S. R. Wickramasinghe, N. Soice, S. M. Husson, *J. Membr. Sci.* **2008**, *311*, 225.

[59] L. Guo, P. H. Santschi, in *Environmental Colloids and Particles: Behaviour, Separation and Characterisation*, Vol. 10 (Eds: K. J. Wilkinson, J. R. Lead), John Wiley & Sons Ltd, West Sussex, England 2007, 159.
